# Triterpenoids as Reactive Oxygen Species Modulators of Cell Fate

**DOI:** 10.1021/acs.chemrestox.1c00428

**Published:** 2022-03-21

**Authors:** Taotao Ling, Lucinda Boyd, Fatima Rivas

**Affiliations:** Department of Chemistry, Lousiana State University, 133 Chopping Hall, Baton Rouge, Louisiana 70803, United States

## Abstract

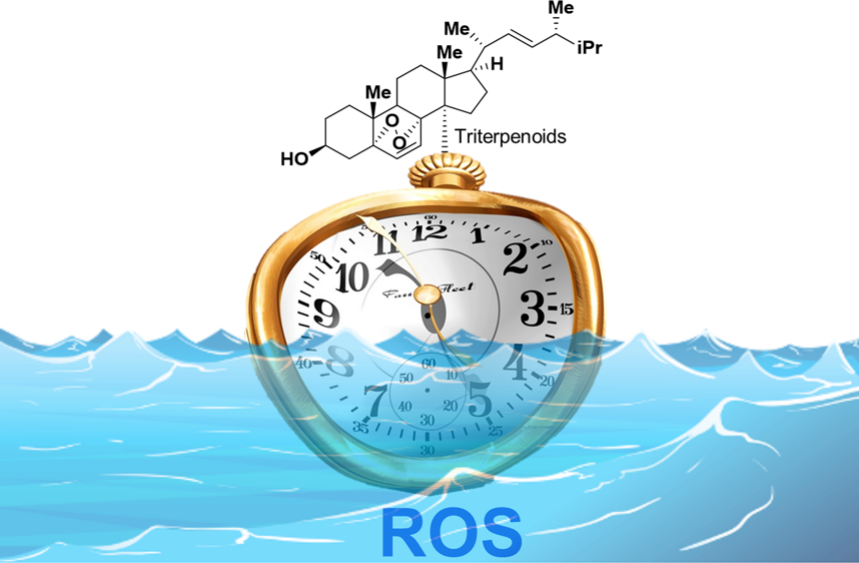

The triterpenoid natural products have played an important role in understanding mechanistic models of human diseases. These natural products are diverse, but many have been characterized as reactive oxygen species (ROS) modulators. ROS can regulate cell survival and function, which ultimately affects biological processes leading to disease. The triterpenoids offer an untapped source of creativity to generate tool compounds with high selectivity to regulate ROS. This brief Review highlights the diverse complexity by which these secondary metabolites induce many cell death modalities (apoptosis, autophagy, ferroptosis, etc.) that can affect various complex cell signaling pathways through ROS and ultimately lead to evading or accelerating cell death.

## 1 Introduction

Natural products and their structural analogues are major contributors to our current pharmacophore repertoire against cancer and infectious diseases.^1^ While natural products offer opportunities for drug discovery, technical challenges such as isolation and structural elucidation remain as barriers in current drug discovery platforms of the pharmaceutical industry. Nonetheless, technological advancements including improved analytical tools, microbe genome mining, and engineering strategies have promoted the ongoing efforts in the area of triterpenoid chemistry.

**Figure 1:**
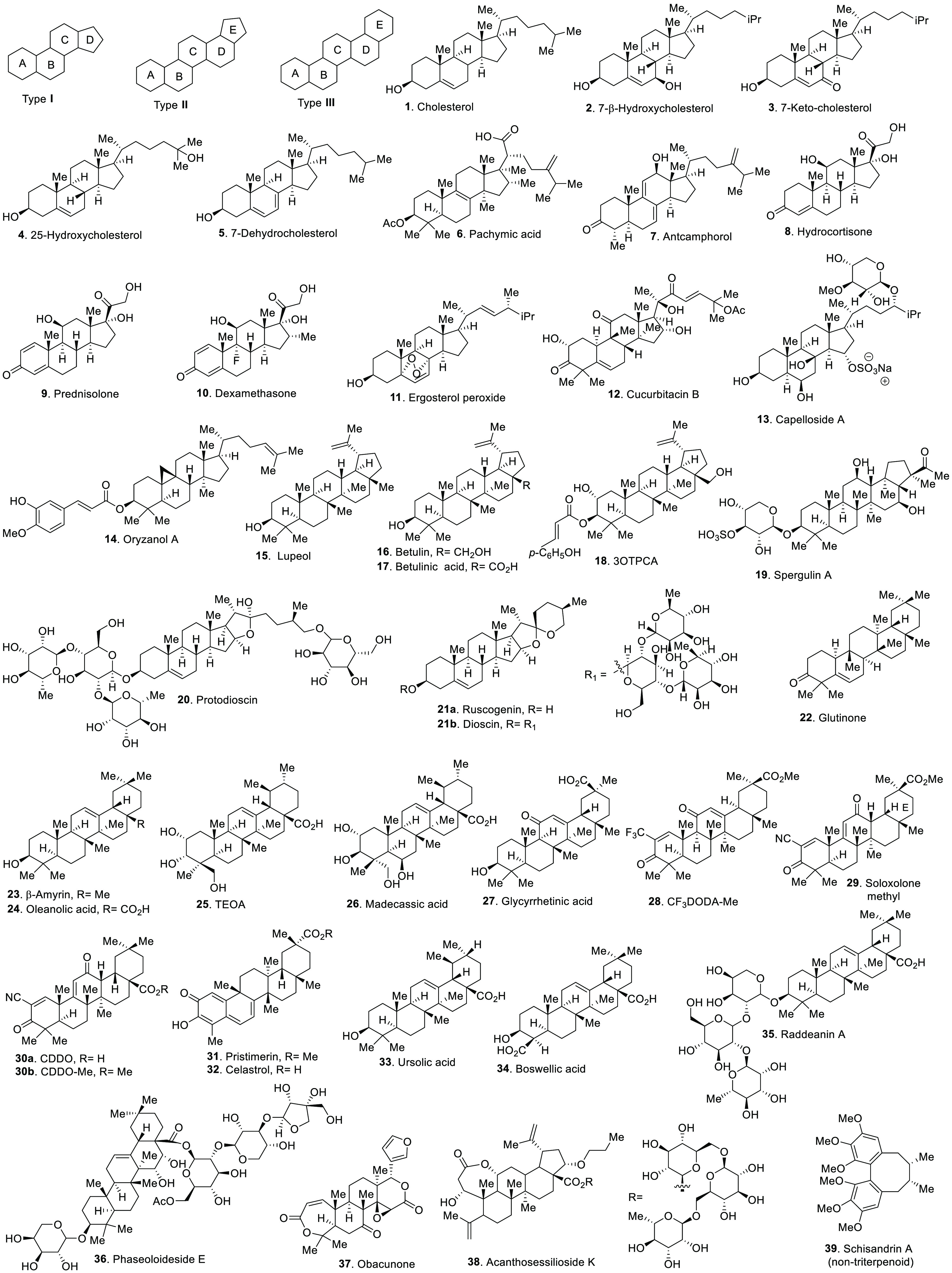
Selected bioactive triterpenoid natural products and/or derivatives.

Terpenoids are the largest class of natural products, most of which are derived from plants but are found in all classes of living organisms serving as energy sources, biological building blocks, and/or signaling molecules.^2^ The terpenoids are grouped in three classes: sesquiterpenoid, diterpenoids, and triterpenoids.^3^ The higher complex triterpenoids and steroidal structures have been recognized for their biological functions as they can display various roles in cell signaling and/or act as receptor ligands. This short Review highlights recent studies of triterpenoids with promising biological properties that allow for an in-depth mechanistic understanding of their mode of action.^4^ Triterpenoids known for regulating various biological actions through their antiproliferative, antiangiogenic, antimetastatic, and apoptotic activities are the most widely studied. In terms of complexity, the triterpenoid natural products can be further categorized into three molecular scaffolds, specifically, type I triterpenoids **1**–**14**, type II triterpenoids **15**–**21**, and type III triterpenoids **22**–**36** (type I–III, Figure 1), featuring the fused ABCD or ABCDE ring systems with various degrees of unsaturation, late-stage oxidation, alkylation, and/or glycosylation (compounds **1**–**39**, Figure 1).

Therefore, triterpenoids serve as a diverse and vast platform of inspiration for medicinal chemists in search of developing novel chemical entities toward clinical candidates. Particularly, these triterpenoid compounds can serve as inducers or inhibitors of reactive oxygen species (ROS), enabling in-depth mechanistic studies of various cellular signaling pathways from the membrane-required component cholesterol **1** to the life-saving agent dexamethasone (compound **10**) and many other steroids. These triterpenoids show distinctive conformations that enable them to display such an array of bioactivities.

This Review summarizes recent findings of triterpenoid natural products and their derivatives on signaling pathways involving master regulators of cell fate, e.g., ERK1 and ERK2 mitogen-activated protein kinases, stress-activated protein kinase 1c, and nuclear factor-kappa B (NF-kB), which are directly or indirectly regulated by ROS, highlighting the therapeutic potential of this family of complex triterpenoid natural products.

## 2 Impact of ROS on Human Health

**Figure 2:**
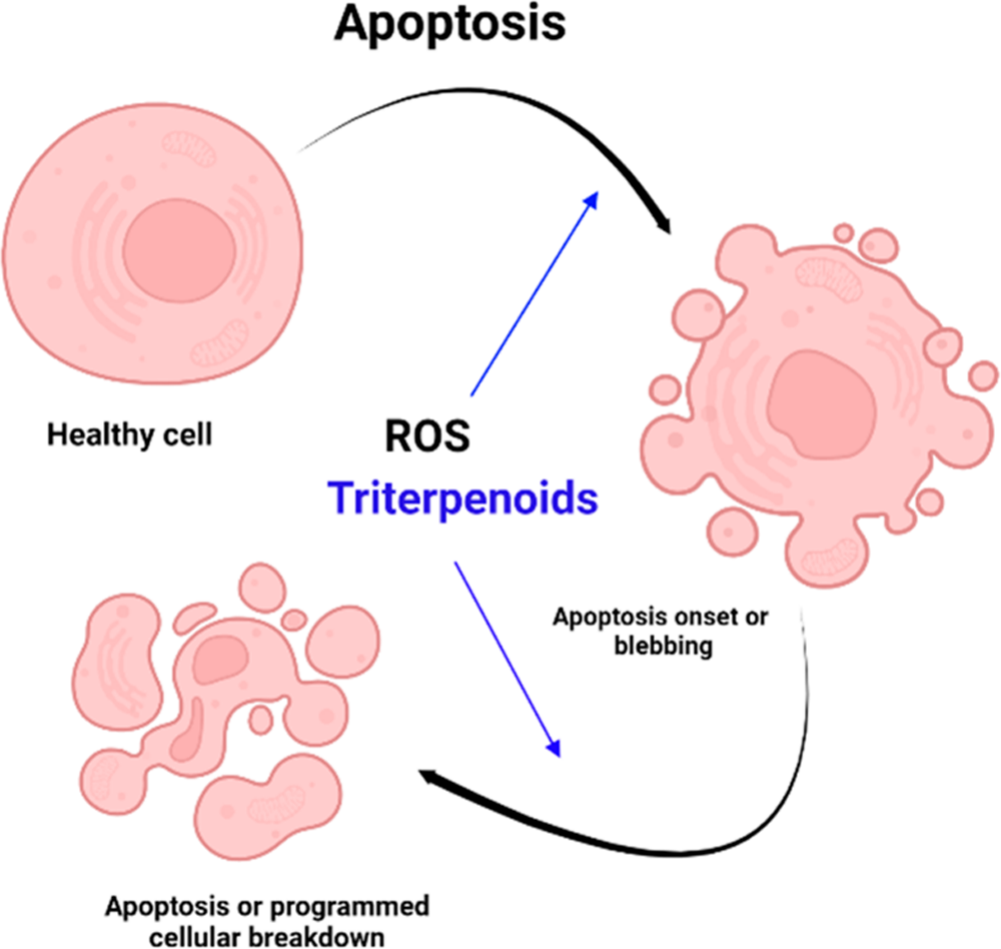
Programmed cell death induced by triterpenoid treatment.

ROS play essential roles in pathophysiologic processes mediating cellular homeostasis. ROS are implicated in several cellular functions from basic signaling to complex communications involved in metabolic processes, oxidative stress modulation, inflammatory response, or cell death. ROS will impact a system depending on various environmental factors (such as temperature and concentration) in a time-dependent manner. ROS are generated in response to a wide variety of internal or external stimuli and support intracellular signaling. The resultant signal will vary depending on the type of ROS formed in the biological system, and they include peroxide, hydroxyl, nitric oxide, singlet oxygen, nitrogen dioxide, and peroxynitrite radicals.^5^ Triterpenoid natural products can modulate a cascade of events through ROS-mediated processes. Excessive disruption of relevant cellular pathways will turn on a signaling cascade for apoptosis, while on the other hand, they can also exert the antioxidant power to quench ROS. Disbalance in mitochondrial proteins such as BCL-2 (B-cell CLL/lymphoma 2, which promotes the release of key factors responsible for apoptosis) can activate caspase mediated cell death^6^ as depicted in Figure 2.

**Figure 3:**
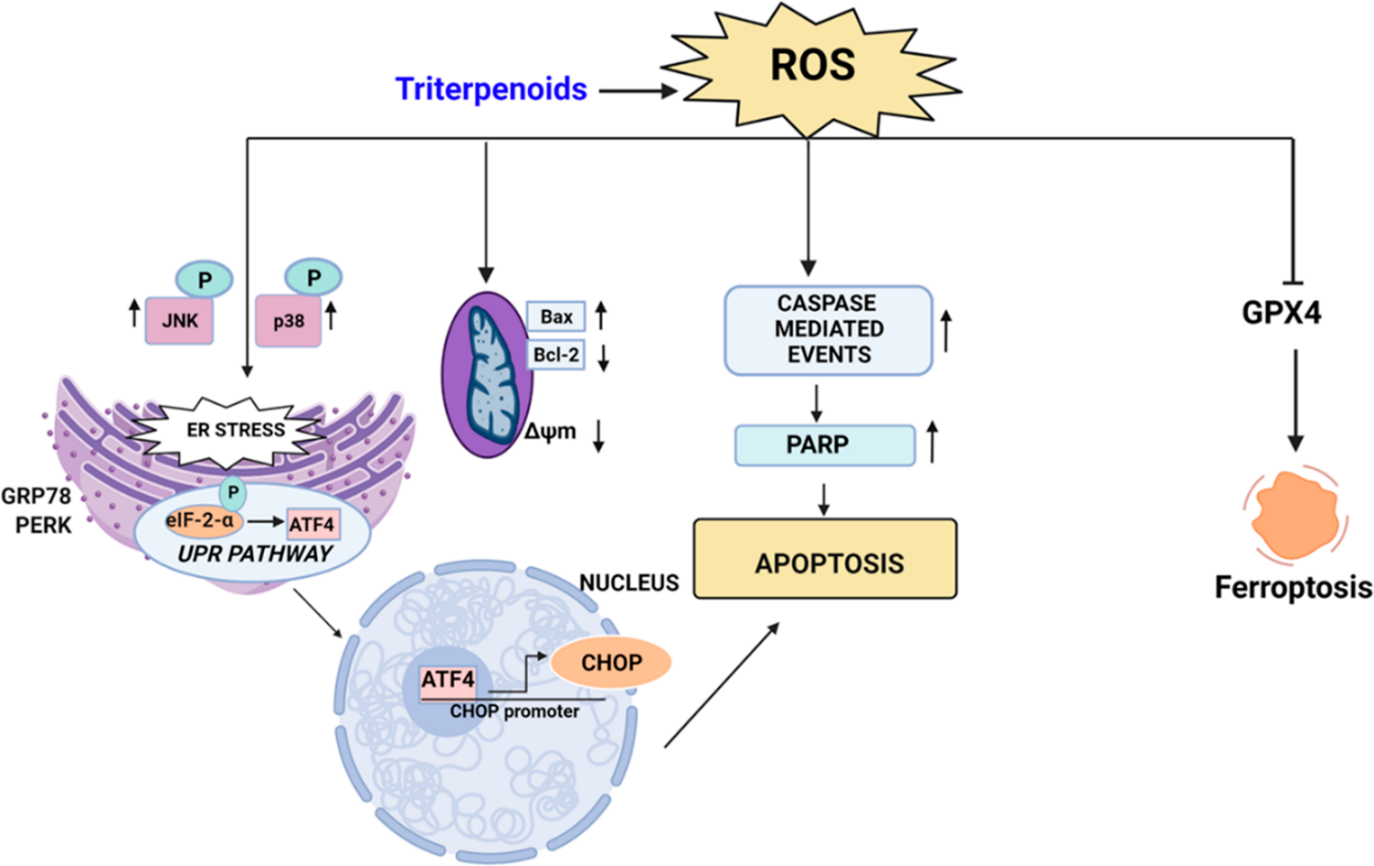
Triterpenoids modulate cell fate via ROS.

Triterpenoids are also responsible for modulating other cell death modalities such as autophagy and ferroptosis. ROS have been directly implicated in these cellular processes using different analytical tools.^7^ Autophagy is a conserved cellular degradation and recycling process that occurs in the lysosome and is vital for cellular homeostasis. However, its dysfunction is associated with a variety of human pathologies, including aging, cancer, neurodegenerative disorders, and heart and metabolic diseases, such as diabetes.^7^ Alternatively, ferroptosis (Figure 3), a nonapoptotic death modality, is characterized by intracellular accumulation of lipid peroxidation and is suppressed by iron chelators or lipophilic antioxidants. Ferroptosis has been associated with acute kidney injury, cancer, and cardiovascular, neurodegenerative, and hepatic diseases.^8^ Within a mammalian cell, the mitochondrion is a major contributor of ROS production, and therefore a cell-fate decision-making organelle. Nonetheless, other cellular organelles such as the endoplasmic reticulum (ER) also play important roles in ROS action. ROS can also lead to the activation of the unfolded protein response (UPR) pathway, a process that adjusts the protein folding ability of the ER to maintain cellular homeostasis as illustrated in Figure 3.

Triterpenoids can lead to the activation of the UPR pathway through master regulators responsive to stress stimuli, such as JNK/P38 (Jun N-terminal kinase and p38 mitogen-activated protein kinase) or ATF4 (activating transcription factor 4), which results in apoptosis. Alternatively, triterpenoids can also directly disrupt the mitochondrial membrane potential and induce caspase 3/9 (intrinsic) or caspase 8 (extrinsic) apoptosis via ROS,^6^ and the most recently identified cell death modality, namely ferroptosis, involves GPX4 (glutathione peroxidase 4) inhibition and/or lipid peroxidation (Figure 3).^8^

Total cellular ROS or mitochondrial-specific ROS at basal and dynamic levels throughout long-term treatments can be monitored by flow cytometry and spectrofluorometry. Several chemical probes to detect ROS have been developed.^9−11^ The scientific investigation of the biological importance of ROS signals requires sensitive and specific tools to allow spatial and quantitative analysis.^11^ However, ROS present in biological systems pose several challenges such as their relatively short lifetime, low concentration, and interactions with several native cell antioxidants that exist *in vivo*, impairing ROS detection and measurements. Thus, dynamic chemical probes with the potential for spatial and temporal specificity of ROS generation will provide highly relevant information to overcome some of the current limitations, which might impair detection and measurement. ROS are implicated as metabolite initiating factors in host cell damage under conditions leading to oxidative stress. The ROS signaling is not likely to migrate a great distance from where they are generated. The spatial and temporal specificity of ROS generation will provide highly valuable information regarding the exact physiological roles of ROS. Furthermore, as intracellular signaling molecules, ROS are critical for innate immune defense against certain microbial pathogens to maintain cellular function.^12^

The glycosylated steroidal triterpenoid molecules have shown promising pharmacological activity. For instance, protodioscin (compound **20**, Figure 1) has demonstrated cytotoxic effects against several cancer cell lines.^13^ The compound disrupts mitochondrial membrane potential and induces intracellular ROS generation and ER stress. The mechanistic studies of protodioscin have shown that it induced apoptosis via caspase-8, -3, and -9, PARP1 (poly(ADP-ribose) polymerase), and BAX (BCL-associated X, an apoptosis regulator), all indicators of programmed cell death, and downregulation of BCL-2 expression. It also induced ROS, which could be quenched by pretreatment with the antioxidant *N*-acetyl cysteine (NAC). Compound **20** also activated the ER stress pathway through induction of ATF4 (activating transcription factor 4) and CHOP (C/EBP homologous protein). The the latter belongs to the family of CCAAT/enhancer binding proteins (C/EBPs) and regulates genes that encode proteins involved in proliferation, differentiation, and energy metabolism. Further studies are needed to better understand the mode of action of this compound.^13^ The related natural product, dioscin (compound **21b**, Figure 1), also induced ROS in gallbladder cancer cell models, leading to oxidative damage and ultimately cell death.^14^ Compound **21b** induced cell migration inhibition and cytotoxicity as measured by phosphorylation of PARP1 and the phosphatidylinositol-3-kinase/protein kinase B (PI3K/AKT) signaling pathway (which regulate cellular processes involved in cell growth, proliferation, metabolism, motility, survival, and apoptosis). Aberrant activation of the PI3K/AKT pathway promotes the survival and proliferation of tumor cells in many human cancers, hence its importance to study their regulation in relevant cellular model systems. However, dioscin’s mode of action differs from compound **20** since neither antioxidant NAC nor glutathione (GSH) could rescue the cells from generating ROS.^14^ The natural product 2α,3α,24-thrihydroxyurs-12-en-28-oic acid (compound **25**, Figure 1) isolated from the roots of *Actinidia eriantha* showed multiple cell death modalities through ROS induction, which could be attenuated by NAC pretreatment of SW620 cells, a colon cancer cell model. It induced apoptosis through cleavage of caspase-9 and PARP1.^15^ Compound **25** promoted the phosphorylation of PERK (protein kinase R (PKR)-like endoplasmic reticulum kinase) and elF2α (eukaryotic translation initiation factor 2 subunit alpha). These proteins are involved in the biological process by which mRNA is properly translated into proteins in eukaryotes, followed by upregulation of the downstream protein CHOP, suggesting the involvement of the PERK/eIF2α/CHOP signaling pathway and ER stress in the SW620 cells. In addition, the study found that compound **25** also induced mitophagy (the selective degradation of damaged and dysfunctional mitochondria by autophagy) by increasing the expression of proteins directly related to mitochondrial quality control mechanisms (PINK1/PARKIN/p62: PTEN-induced kinase 1, parkinson disease protein 2, ubiquitin-binding protein/p62).^15^ These findings agreed with previous studies that have shown ROS to be an essential element in autophagy and mitochondrial dysfunction and may be instrumental to mitophagy.^16^ Compound **25** demonstrated an ample therapeutic window, offering a potential new lead compound against colon cancer.^15^

Most triterpenoids have been studied as ROS inducing agents, while less is known about their antioxidant or protective effects. Recent studies have demonstrated promising results including findings from triterpenoid saponins, namely, the triterpenoids of *Ilex cornuta*, which have shown to exert cardioprotective effects in rat models of myocardial ischemic injury models such as the hydrogen peroxide (H_2_O_2_) treated H9c2 cardiomyocyte model.^17^ Although, the studies were conducted with the *Ilex cornuta* extract, the mixture inhibited EZH2 (enhancer of zeste 2 polycomb repressive complex 2 subunit, involved in histone methylation and, ultimately, transcriptional repression) activity through the Akt pathway, which is responsible for promoting growth and survival in response to extracellular signals, thereby protecting the H9c2 cells from apoptosis.^17^

The pentacyclic triterpenoid, lupeol (compound **15**, Figure 1), naturally found in several medicinal plants, has shown antioxidant, antineuroinflammatory, and antiamyloidogenic effects.^18^ Using an Aβ-mouse model of Alzheimer’s disease (AD), the study shows that lupeol reverses the memory deficits, and ROS levels were significantly decreased by compound treatment. Therefore, lupeol may serve as a protective agent against Aβ-induced oxidative stress-mediated neuroinflammation, AD, and cognitive dysfunction.^18^ 3-*O*-Trans-*p*-coumaroyl-alphitolic acid (3OTPCA, compound **18**, Figure 1), a triterpenoid isolated from the plant *Zizyphus jujuba*, induces apoptotic cell death in human leukemia cells. 3OPTCA induces DNA fragmentation within 24 h after treatment in B and T leukemia cell lines (U937, Molt-4 and Jurkat).^19^ To better understand the underlying mechanisms, the authors performed RNA-seq analysis, which revealed the UPR pathway was upregulated after 3OTPCA treatment. To validate ER stress, thapsigargin, an endoplasmic Ca^2+^ transport ATPase inhibitor, was used as a comparison to 3OTPCA. Both compounds increased intracellular calcium levels, downregulated the expression of BCL-2, and led to the loss of the mitochondrial membrane, indicating UPR activation. Moreover, 3OTPCA induced superoxide anion generation and caspase-8 cleavage without affecting FAS (cell surface death receptor) expression. The combined studies indicate that 3OTPCA induces apoptotic cell death through activation of UPR and the generation of ROS.^19^

Glycyrrhetinic acid (compound **27**) derivatives have been generated to evaluate their potential as inducers of human cancer cell death, and the derivative soloxolone methyl (compound **29**, Figure 1), bearing a cyano enone functionality at the A ring, has shown the most potency against human cervical carcinoma KB-3-1 cell models.^20^ Compound **29** affected several cellular proliferative and survival pathways including protein–protein interaction networks involved in ER stress. Furthermore, using various connectivity analysis programs for transcriptomic data, compound **29** showed a similar behavior to known ER stress inducers such as thapsigargin and geldanamycin, suggesting the biological targets might be SERCA (sarcoendoplasmic reticulum calcium ATPase, responsible for maintaining cytosolic calcium levels to mediate an array of signaling pathways and physiological processes) and HSP90B1 (heat shock protein 90 beta family member 1), an important chaperone for protein folding and quality control in the ER. Both proteins are promising biological targets for drug discovery. The findings indicate this family of triterpenoid molecules offer promising hit compounds for the treatment of selected cancer subtypes that are dependent on proper protein folding.^20^

Compounds isolated from *Antrodia camphorate*, a medicinal mushroom, which include the lanostane-type triterpenoids also known as antcamphorols (representative compound **7**, Figure 1), showed promising biological activity. The release of ROS has been associated with various cardiovascular diseases and may play a role in vascular complications of diabetes. Using high-glucose-induced oxidative human umbilical vein endothelial cells (HUVECs) as an injury cell model, the compounds showed significant ROS scavenging activity in a dose-dependent manner. Furthermore, the antcamphorol compounds did not show significant cytotoxicity against normal cells, indicating its therapeutic potential against vascular inflammation and dysfunction.^21^

**Figure 4:**
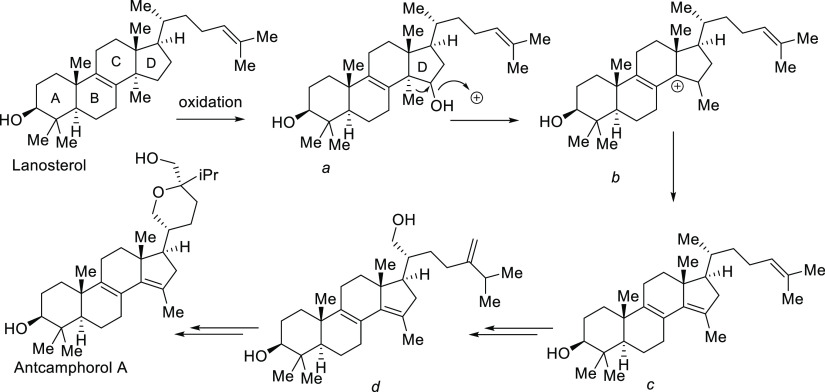
Biosynthesis of antcamphorol A.

From a structural perspective, the molecular scaffold of triterpenoids can be biosynthetically derived from related family members. Terpenes originate from the C_5_ substrates dimethylallyl diphosphate (DMAPP) and isopentenyl diphosphate (IPP), typically by initially condensing DMAPP with one or more IPP molecules in a 1′–4 or “head-to-tail” fashion to form (C_10_) geranyl diphosphate, (C_15_) farnesyl diphosphate, or (C_20_) geranylgeranyl diphosphate.^22^ Farnesyl diphosphate and geranylgeranyl diphosphate can then condense in a “head-to-head” mode or tail-to-tail addition to form precursors such as squalene to complex sterols.^22^ While these precursors undergo a series of reactions mediated by enzymes, such as late-stage oxidations, carbocation mediated alkyl shifts, and intramolecular cyclization reactions, a representative for antcamphorol triterpenoid was recently reported.^22^ A plausible biosynthetic pathway of the triterpenoid family member antcamphorol was proposed as depicted in Figure 4. Starting with the hydroxylation of the D-ring via bio-oxidation, followed by a unique acid-promoted methyl-migration and the consequent olefin formation, the molecular scaffold of antcamphorol can be derived from its precursor lanosterol, and the late-stage oxidation and cyclization of the D-ring side chain of the advanced intermediate (species d, Figure 4) furnished the tetrahydrofuran moiety of antcamphorol triterpenoid.^21^

A class of lignans and triterpenoids from the genus *Schisandra* has been reported to display antioxidant properties. One of the compounds from this plant, the nontriterpenoid schisandrin A (compound **39**, Figure 1), was shown to decrease DNA damage and apoptosis in the myoblast cellular model C2C12 after H_2_O_2_ treatment. The study showed that schisandrin A prevented the release of cytochrome c, presumably by protecting the mitochondria from the oxidative effects of H_2_O_2_. Therefore, schisandrin A may have a beneficial effect on the prevention and treatment of diseases associated with oxidative stress.^23^

Inflammatory conditions are typically associated with oxidative stress led by ROS or hyperactivation of other mediators.^12^ Acute inflammation is a protective immune response led by the innate immune system in response to harmful external or internal stimuli, while chronic inflammatory diseases arise from prolonged insult such as neurodegenerative diseases (multiple sclerosis, Alzheimer’s disease, and Parkinson’s disease) and metabolic disorders (type 2 diabetes and obesity). Studies have shown that natural products can modulate immunomodulatory action, targeting important modulators of inflammation such as TNF-α, IL-1β, and nitric oxide (NO) production, indicating a potential avenue for new anti-inflammatory agents.^24^ A mixture of plant esterified sterols (with ferulic appendages and triterpene alcohols, collectively known as γ-oryzanol, has been reported to display such unique properties. This mixture has shown potent antioxidant activity and can modulate lipid metabolism in a H_2_O_2_ human hepatic L02 cell model.^25^ Overproduction of ROS can induce cellular oxidative damage, and a widely acceptable model is the treatment of cells with peroxides such as *t*-butyl peroxide (*t*-BHP) or H_2_O_2_ to induce oxidative injury. H_2_O_2_ treatment increases in levels of malondialdehyde (MDA) and ROS, decreases superoxide dismutase (SOD) and catalase (CAT) activity, induces the loss of mitochondrial membrane potential, and increases protein expressions of caspase-3 and -9, leading to apoptosis. Pretreatment of the cells with γ-oryzanol enhanced the ROS scavenging activity of endogenous antioxidant enzymes (SOD, CAT) and decreased lipid peroxidation and apoptosis by restoring mitochondrial membrane potential, indicating that γ-oryzanol can suppress intracellular accumulation of ROS and prevent ROS-activated mitochondrial apoptotic pathways. However, it is unlikely that this compound mixture can rescue the cells once ROS has commenced, as pretreatment of the cells with γ-oryzanol was required to observe a quantifiable effect.^25^ In parallel, the investigation of compound **16** showed that pretreatment of mammalian cells with compound **16** prior to *t*-BHP exposure significantly inhibits intracellular ROS accumulation when compared with controls.^26^

Madecassic acid (compound **26**, Figure 1), an abundant triterpenoid found in *Centella asiatica* (L.) Urban has shown an antioxidative effect in periodontitis, an inflammatory disease.^27^*In vitro* experiments indicate that compound **26** prevents H_2_O_2_-induced oxidative stress and apoptosis in human periodontal ligament fibroblast (hPDLF) cells, an important cellular model to identify compounds against inflammatory disease, by reducing intracellular ROS production and maintaining mitochondrial membrane potential (ΔΨ_m_). Therefore, the administration of compound **26** may be an alternative therapeutic approach to repair cell damage in periodontitis.^27^

A recent study identified glucocorticoids (such as compounds **8**–**10**, Figure 1) as inhibitors of mitochondrial superoxide production in microvascular endothelial cells exposed to elevated extracellular glucose, which minimized ROS damage.^28^ Glucocorticoids induce expression of the mitochondrial UCP2 (uncoupling protein 2). The proton gradient across the inner mitochondrial membrane is a key driving force for mitochondrial ROS production, and this gradient can be modulated by members of the mitochondrial UCP family and affect the overall mitochondrial membrane potential. To further validate their findings, the study found that UCP2 silencing prevents the protective effect of the glucocorticoids on ROS production. The function of UCP2 can be quickly regulated via glutathionylation during oxidative stress, enabling UCP2 to directly dial ROS levels. Therefore, UCP2 induction might represent a novel experimental therapeutic intervention in diabetic vascular complications. Repurposing glucocorticoids as therapeutics may pose challenges due to glucocorticoid side effects during chronic administration. These findings suggest the development of selective modulators of the UCP2 pathway as a potential treatment for diabetic vascular complications should be considered.^28^ Another natural product with promising antioxidant properties is betulin (compound **16**, Figure 1).^29^ Betulin has displayed potent effects against inflammatory cells by reducing ROS generation, increasing antioxidant enzyme expression, and attenuating the level of oxidative markers in an ovalbumin-induced mouse model of asthma. The study showed treatment with compound **16** downregulated the expression of genes involved in remodeling during inflammation such as MMP-9 (matrix metallopeptidase 9), *t*TG (tissue transglutaminase), and TGF-β1(transforming growth factor beta 1). Compound **16** also downregulated TREM-1 (triggering receptor expressed on myeloid cells-1), p-IκB-α (nuclear factor of kappa light polypeptide gene enhancer in B-cell inhibitors), and NF-κBp65 protein levels in the lungs of the mice. Furthermore, the levels of interleukin (IL) cell signaling proteins (IL-4, IL-5, and IL-13) were significantly reduced by treatment with **16** in a similar pattern to the dexamethasone-treated group of animal models.^24^ These cytokines regulate the inflammatory response in order to clear foreign entities by changing the abundance of various cell populations through binding to their corresponding cellular receptors. The combined studies indicate the betulin compounds might be complementary to current anti-inflammatory treatments.^29^

**Figure 5:**
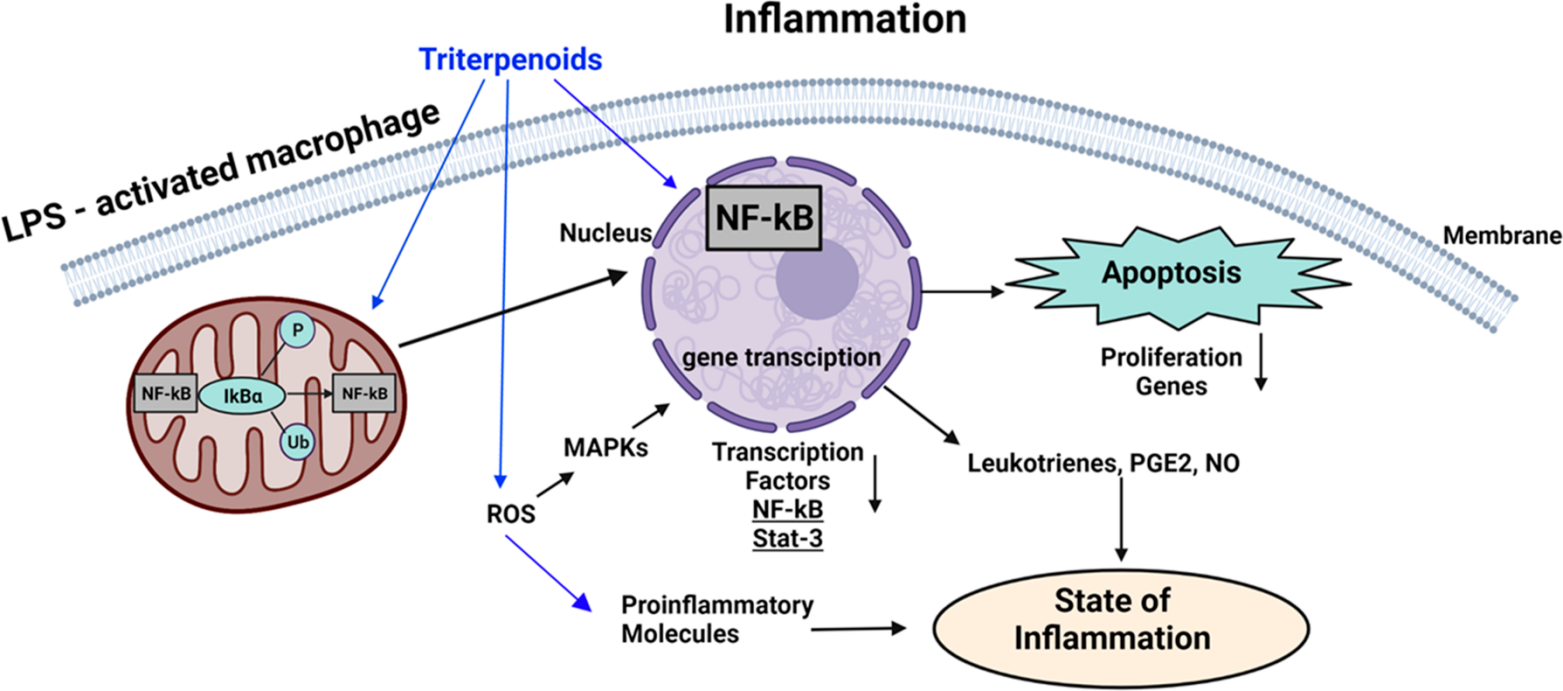
Triterpenoids and inflammation.

Raddeanin A (compound **35**, Figure 1) isolated from *Anemone raddeana Regel* displayed anticancer properties in *in vitro* and *in vivo* osteosarcoma cancer models. Compound **35** induced mitochondria-dependent apoptosis via ROS induction and suppressed metastasis *in vitro*. Treatment with compound **35** decreased p-IκBα and reduced p65 levels in the cellular models, which is associated with inhibition of NF-κB transcriptional activity, leading to cancer cell death. As illustrated in Figure 5, the NF-κB family of transcription factors is a master regulator of immune development, immune responses, inflammation, and cancer. The NF-κB signaling system defined by the interactions between NF-κB dimers, IκB regulators, and IKK complexes is responsive to several cellular stimuli, and upon receptor–ligand engagement, specific cellular actions will be activated. Triterpenoids have been reported to have effects on all branches of this complex signaling pathway by mediating apoptosis through mitochondria action or MAPKs (mitogen-activated protein kinases) regulation. Triterpenoids are modulators of the NF-κB pathway, as the cell state of inflammation can be reduced through this signaling pathway.^12,30^ Another important factor in inflammation is STAT3, which belongs to the STAT (signal transducer and activator of transcription) family of signal responsive transcription factors, which are kept in an inactive form in the cytoplasm of nonstimulated cells in a similar manner as NF-κB. Once STAT3 is activated, it can regulate the expression of a variety of genes in response to cellular stimuli and thus plays a key role in cell growth and apoptosis (Figure 5). The triterpenoid celastrol (compound **32**, Figure 1) has been recently shown to inhibit Ang II-induced cardiac dysfunction by inhibiting STAT3 activity in *in vitro* and *in vivo* models.^30^ Furthermore, at low concentrations, compound **35** inhibited the migration and invasion of osteosarcoma cells by suppressing MMP-2/9 expression, which is also dependent on NF-κB transcription. To validate the mode of action of compound **35**, silencing of p65 in these cell lines was conducted, and treatment with compound **35** increased the sensitivity of the osteosarcoma cells during migration and invasion assays, validating compound **35** as a ROS modulator of the NF-κB signaling pathway. Because human osteosarcoma has a high mortality in adolescents with a poor prognosis due to the high incidence of metastasis, these findings offer a potential lead compound against this cancer subtype.^31^

The natural products 3,4-seco-triterpenoid glycosides, including acanthosessilioside K (a representative of their large family, compound **38**, Figure 1) from *Acanthopanax sessiliflorus* (*Araliaceae*) fruits, have been reported to deactivate or reduce the expression of inflammation markers (NO, PGE2, TNF-α, IL-1β, IL-6, iNOS, and COX-2) in lipopolysaccharide (LPS)-activated BV2 and RAW264.7 murine cells. The acanthosessilioside triterpenoids are large molecules highly decorated with sugars, which improves their solubility properties, rendering them as promising preclinical candidates for further development against inflammatory and neuro-inflammatory disorders.^32^ Other related steroidal glycosides with potential therapeutic value are the natural products isolated from the starfish *Ogmaster capella* (capelloside A, coscinasteroside B), which have been shown to reduce intracellular ROS levels in the LPS-activated murine macrophage RAW264.7 cell line, while showing little to no cytotoxicity toward normal cells at concentrations up to 100 μM, indicating the therapeutic index.^33^

Most inflammatory conditions are associated with oxidative stress and/or hyperactivation of COX mediators. Glutinone (compound **22**, among other similar natural products, Figure 1) was isolated from the plant *Scoparia dulcis* and tested for its immunomodulatory potential by an oxidative burst assay.^34^ Compound **22** showed a significant inhibitory effect on the release of ROS from zymosan activated cells and isolated polymorphonuclear leukocytes as compared to the standard drug, ibuprofen. In addition, compound **22** was found to moderately inhibit the pro-inflammatory cytokines TNF-α and IL-1β and NO production. Therefore, further *in vivo* model studies of compound **22** are required to understand its mode of action in inflammatory diseases.^34^ Recent studies have shown that spergulin-A (compound **19**, Figure 1) showed enhanced production of intracellular NO and ROI (reactive oxygen intermediates) in leishmania models. Parasite infection is responsible for the suppression of macrophage microbicidal activity that relies upon NO, ROI, and cytokines like IL-1, IL-12β, and TNF-α. Treatment with compound **19** enhanced NO production and overall parasite control by cytokine modulation.^35^

Glycyrrhizin acid (compound **27**, Figure 1), bardoxolone (CDDO, the free carboxylic acid, compound **30a**), bardoxolone-methyl (Bar-Me, compound **30b**, Figure 1), and methyl 2-trifluoromethyl-3,11-dioxoolean-1,12-dien-30-oate (CF_3_DODA-Me, compound **28**) are structurally related pentacyclic triterpenoids, featuring 2-cyano-1-en-3-one or 2-trifluoromethyl-1-en-3-one moieties in the A-ring (Figure 1), and differ in the position of the enone system in the C ring. Compound **30b** forms a Michael addition adduct with GSH and inhibits IKKβ phosphorylation, while no adduct was observed with compound CF_3_DODA-Me (compound **28**), presumably due to the steric hindrance provided by the C ring.^36^ Therefore, compound **30b** is a much more reactive electrophile than compound **28** toward thiol-containing proteins, providing compound **28** with a more ample therapeutic index.^36^ However, both compounds induced ROS, inhibited cell growth, induced apoptosis, and decreased expression of the specificity proteins (Sp) and c-MYC in various cancer cell models lines.^36^ Compound **28** also exerted antioxidant, anti-inflammatory, and anticancer activities in several cell lines via a DNA damage response, which increases cellular levels of the antioxidative enzymes heme oxygenase-1 (HO-1), NAD(P)H dehydrogenase (quinone 1), and mitochondrial superoxide dismutase 2.^37^ Compound **30b** increased intracellular ROS levels in human peripheral blood mononuclear cells (PBMCs). However, in radiation-induced DNA double strand break (DSB) formation, the γ-H2AX+53BP1 DSB foci assay and the cytokinesis-block micronucleus assay pretreatment with nanomolar compound **30b** of the PBMCs did not affect γ-H2AX+53BP1 DSB foci formation or the frequency of micronuclei, indicating the pharmacological increase of antioxidative enzymes might not mitigate the results of radiation in the PBMCs due to the short-term compound **30b** treatment or the rapid consequences of ROS damage. Further studies on the antioxidative proteins involved in stress-induced hyper-metabolism during a DNA damage response are warranted.^37^ Compound **28** also induced ROS and inhibited telomerase activity in MiaPaCa-2 and Panc-1 pancreatic cancer cell lines. Pretreatment with NAC blocked the telomerase inhibitory activity and inhibited the expression of several human telomerase reverse transcriptase (hTERT) regulatory proteins (e.g., c-MYC, SP1, NF-κB, and p-AKT), indicating the observed biological activity is mediated through ROS-dependent mechanisms.^36,37^

Compound **30** induced ROS in rhabdomyosarcoma (RMS) cell models (RD and Rh30), leading to apoptosis as well as inhibited growth and invasion in RMS cells.^38^ The compound response was attenuated after cotreatment with the antioxidant glutathione, indicating its anticancer activity is driven by ROS. Compound **30** downregulated the expression of key proteins associated with cell proliferation (cyclin D1 and multiple receptor tyrosine kinases), cell survival (survivin and BCL-2), angiogenesis/metastatic potential [MMP-9, vascular endothelial growth (VEGF)], and inflammation (NF-κB). The effects of compound **30** further emphasize the sensitivity of RMS cells to ROS inducers and the clinical potential of these compounds to treat this cancer subtype.^38^

CDDO (compound **30a**) is likely to activate NRF2 (nuclear factor, erythroid 2-like 2) through the targeting of reactive cysteine amino acids on KEAP1 (Kelch-like protein 19, a major regulator of redox homeostasis controlling enzymes involved in detoxification and cyto-protection), and it can also interact with additional pharmacological targets, including IKKβ, which modulates NF-kB signaling, and mTOR55 among other important regulators of cell fate. To maximize the properties of CDDO (compound **30a**), a conjugate molecule was generated to improve new technologies such as PROTAC (proteolysis targeting chimeric) to selectively and catalytically degrade their biological target.^36^ Recent studies suggest clinical resistance to PROTAC molecules can occur through rewiring of the cellular E3 ligase machinery, highlighting the critical need for the discovery of diverse E3 ligase recruiters.^36b^ The first selective inhibitor of BRD4 (bromodomain containing 4, an epigenetic protein family member) was a thienotriazolodiazepine also known as JQ1.^39^ Therefore, CDDO-JQ1 was synthesized and led to an efficient proteasome-mediated degradation of BRD4. The reported data demonstrated that CDDO effectively binds the Kelch domain of the KEAP1/Cul3 complex, where the degrader presumably binds, representing a novel bifunctional protein degrader based on the known KEAP1 ligand CDDO interaction.^36^ Epigenetic proteins have emerged as potential biological targets in drug discovery, primarily the chromatin modifying enzymes, also known as epigenetic writers and erasers. This family of epigenetic readers regulates gene expression as components of transcription factor complexes and determinants of epigenetic memory, playing important roles in cell proliferation, pro-inflammatory events, and immune responses.^39b^

In search for more effective therapeutics against pancreatic cancer, one of the least treatable malignancies, a high throughput study found the natural product pristimerin (compound **31**, Figure 1) provided an opportunity to be further developed as an anticancer agent with promising therapeutic potential. In the study,^40^ compound **31**, a quinonemethide triterpenoid, was shown to inhibit the proliferation of pancreatic cancer cell lines by promoting apoptosis characterized by increased Annexin V binding, PARP1, and procaspase-3, -8, and -9. The mechanistic study revealed that compound **31** promoted mitochondrial depolarization and their release of cytochrome c; this study has also suggested the apoptosis-induced pharmacological effect of compound **31** was due to its inhibition of the pro-survival AKT/NF-kB/mTOR signaling pathway and antiapoptotic BCL-2; therefore, the further *in vivo* study of compound **31** as a potential therapeutic agent against this malignancy is warranted.^40^

β-Amyrin (compound **23**, Figure 1) and related natural products cohulupone and garcinielliptone P have been disclosed as ROS modulators and inhibit xanthine oxidase (XO) in a cisplatin-treated NTUB1 (a human bladder cancer cell) cell model.^41^ Furthermore, the treated cancer cells at low doses of compound **23** suffer from cell cycle arrest and apoptosis, indicating the therapeutic potential of these compounds.^41^

Recent studies have shown steroidal compounds such as 25-hydroxycholesterol (compound **4**, Figure 1) are generated by macrophages to regulate an anti-inflammatory circuit that maintains mitochondrial integrity and prevents DNA sensor protein Absent In Melanoma 2 (AIM2) inflammasome activation.^42^ The study found that increasing the macrophage cholesterol content is sufficient to trigger type I interferon restrains interleukin-1β (IL-1β) release in a AIM2-dependent manner, indicating compound **4** can regulate inflammation in chronic diseases like obesity and metabolic syndrome. Previous studies have identified the relationship between cholesterol-dependent mitochondrial dysfunction under high fat conditions and the development of metabolic diseases.^42^ Other oxysterols such as 7-dehydrocholesterol (7-DHC, compound **5**, Figure 1) sharing the cholesterol (compound **1**) core have been reported to act as endogenous lipidic smoothened modulators and may be associated in the pathology of Smith–Lemli–Opitz syndrome, a human disease caused by genetic loss of the enzyme 7-DHC reductase. Further mechanistic studies are required to determine the exact mode of action of their therapeutic potential, but it is important to highlight the diverse roles of steroidal compounds.^43^

## 3 ROS and Other Cell Death Modalities: Autophagy and Ferroptosis

**Figure 6:**
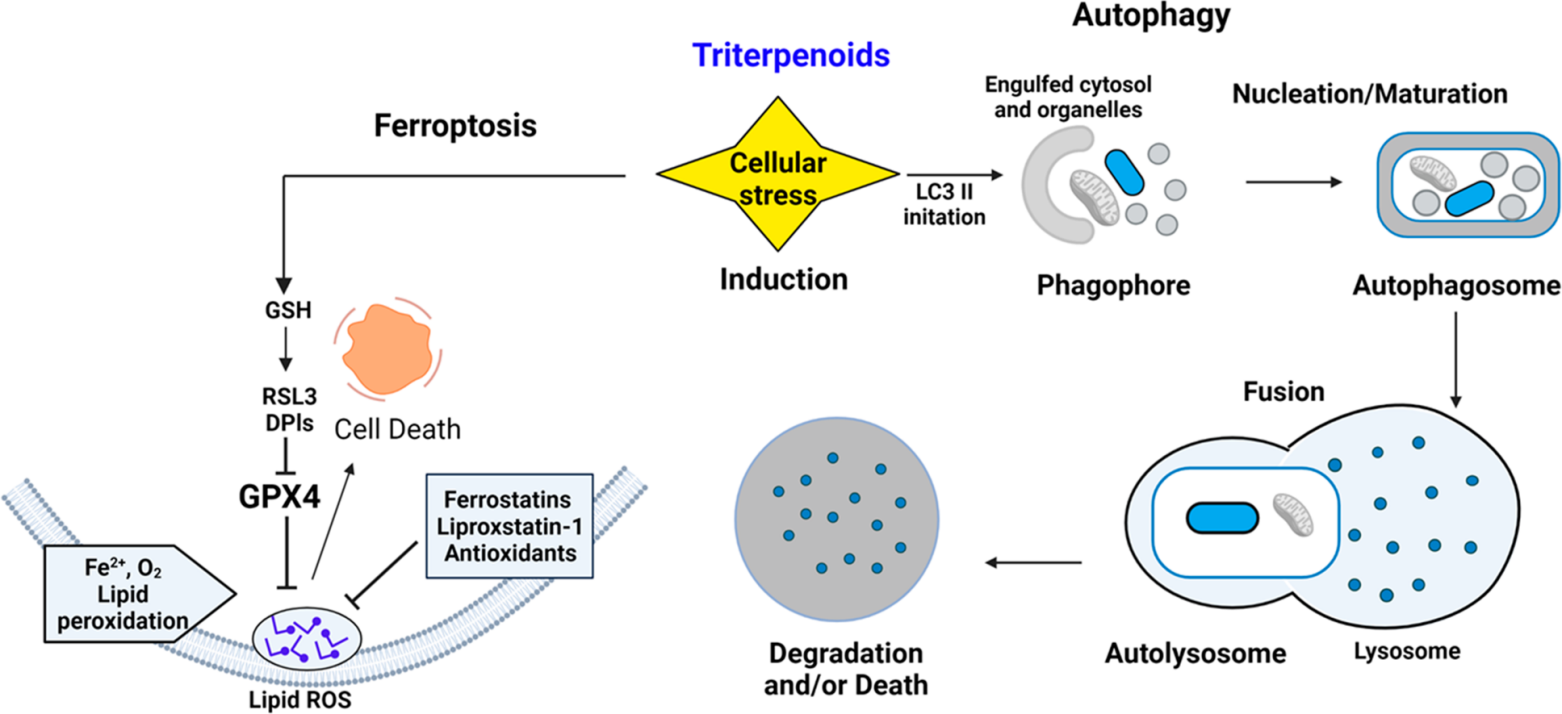
Other cell death modalities influenced by ROS.

Terpene/steroidal molecules can regulate cell death through multiple pathways including apoptosis and various cell death modalities through ROS modulation. Two arising areas are autophagy and ferroptosis (Figure 6). Autophagy is recognized as a critical pathway in the catabolism of cellular constituents, such as protein aggregates (aggrephagy), lipid droplets (lipophagy), and carbohydrates. In mammalian cells, there are different types of autophagy (microautophagy, macroautophagy, and chaperone-mediated autophagy). Several chemical probes have been regenerated to study these cellular events. Autophagy can serve as a survival mechanism, but in some instances, it can lead to cell death. 7-Oxysterols (such as compounds **2** and **3**, Figure 1) have been shown to induce autophagy, leading to cellular lipid accumulation and ultimately cell death.^44^ Exposure to 7-oxysterols induced autophagic vacuole synthesis in the form of increased autophagy marker LC3 (microtubule-associated protein 1A/1B-light chain 3) and LC3-phosphatidylethanolamine conjugate (LC3-II) followed by autophagic vacuole formation as illustrated in Figure 6. 7-Oxysterols also increased ATG5 (autophagy related 5) levels and decreased autophagy degradation, as marked by the induction of p62. Autophagy induction by rapamycin minimized 7-oxysterol-induced dysfunctional autophagy and cell death via the reduction of ROS and lysosomal membrane permeabilization (LMP) as well as cellular lipids. The study demonstrated that autophagy may serve as a protective role in the regulation of oxidized lipid-mediated cytotoxicity to limit necrotic core formation in atheroma progression.^44^ Various studies have implicated dysfunctional ferroptosis in the progression of human diseases, including carcinogenesis, ischemia-reperfusion injury, traumatic spinal cord injury, and neurodegenerative diseases.^45^ Several studies have reported that various compounds can cause cancer cell death through the induction of ferroptosis and can overcome drug resistance. Therefore, ferroptosis induction could become an alternative therapeutic treatment for specific cancer subtypes. Ferroptosis can be triggered by small molecules that target the system Xc inhibitor, erastin, or RSL3, a GPX4 inhibitor.^45^ Interest in ferroptosis has increased as recent studies have shown that ferroptosis inducers may enhance the chemosensitivity of drug-resistant cancer cells toward chemotherapeutic drugs.^45,46^ Ferroptosis-based cancer therapies are expected to overcome the limitations of current traditional therapeutics due to resistance to apoptosis or necrosis. Recently, novel anticancer drugs based on the potential therapeutic opportunities and nonapoptotic features of ferroptosis are being developed.^46^ These studies have advanced the exploitation of novel ferroptosis inducers as a valid approach in the development of antineoplastic drugs.

Obacunone (compound **37**, Figure 1), a triterpenoid extracted from *Phellodendronchinense Schneid* or *Dictamnus dasycarpusb Turcz* plant, showed anti-inflammatory, antineoplastic, antioxidant, and antifibrotic effects, among various pharmacological effects.^47^ However, the mechanism of how compound **37** mediates antifibrotic effects in liver fibrosis models remains unclear. Liver fibrosis is a debilitating human disease, and while various inflammatory pathways are activated, it is known to be accompanied by excessive ROS. Studies of a mouse liver fibrosis model induced by carbon tetrachloride (CCl_4_) treatment and hepatic stellate cells (LX2 cell line treated with TGF-β) were performed to test compound **37**. Obacunone demonstrated potent regulatory effects and suppressed various confirmed pathways of this disease model (TGF-β/P-SMAD signals and the epithelia mesenchymal transformation process) and exerted antioxidant properties by reducing the levels of ROS in both models. The antioxidant effect of obacunone was attributed to the activation of GPX4 and NRF-2.^47^

The triterpenoid cucurbitacin B (compound **12**, Figure 1) has been shown to downregulate the expression of GPX4, which allows for the initiation of ferroptosis in the human nasopharyngeal carcinoma (CNE1) cell model.^48^ Compound **12** promotes the accumulation of iron ions and GSH depletion, which leads to lipid peroxidation via the ferroptosis pathway illustrated in Figure 6. Also, compound **12** exhibited antitumor effects by inhibiting cellular microtubule polymerization, arresting cell cycle, and suppressing migration and invasion in *in vitro* cancer models. More importantly, it showed significant inhibition of tumor progression without causing side effects in *in vivo* models. Therefore, compound **12** has therapeutic applications as a promising natural candidate for the development of ferroptosis-inducing agents against cancer.^48^

The triterpenoid CDDO (compound **30a**, Figure 1), a known inhibitor of heat shock protein 90 (HSP90), was recently shown to modulate ferroptosis through degradation of GPX4. It is possible that compound **30a** can regulate cell death through multiple mechanisms, rendering a more suitable compound to avoid drug resistance in the clinic.^49^ Studies indicate that HSP90 does not bind directly to GPX4, but rather it participates in ferroptosis by regulating the levels of LAMP-2 isoform A, an essential step in this biological process. Therefore, other HSP90 inhibitors may also require further studies to evaluate their potential against ferroptosis, presenting an opportunity to repurpose a clinical candidate.^49^

Pachymic acid (compound **6**, Figure 1), a lanostane type triterpenoid from *Poria cocos*, has been shown to positively affect renal ischemia reperfusion injury in *in vivo* murine models.^50^ Treatment with compound **6** enhanced the protein expression levels of GPX4, solute carrier family 7 member 11 (SLC7A11), and heme oxygenase 1 (HO1) in the kidney. It also increased the expression levels of the NRF2 signaling pathway members and reduced overall renal pathological damage. Therefore, compound **6** is a promising agent with protective effects on ischemia reperfusion induced acute kidney injury in mice by promoting the activation of the NRF2 signaling pathway and upregulating the expression levels of the downstream ferroptosis regulated proteins such as GPX4, which led to a decrease of COX2 (mitochondrially encoded cytochrome c oxidase II), the ferroptosis associated lipid peroxidation protein and a lipid oxidation indicator.^50^

Pancreatic cancer cells are dependent on iron for their uncontrolled and rapid proliferation since iron is required for DNA synthesis. Studies have shown that ruscogenin (compound **21a**, Figure 1), a saponin found in the root of *Ophiopogon japonicus*, significantly repressed cell viability and induced cell death in pancreatic cancer cells *in vitro* in a dose- and time-dependent manner.^51^ The findings were further confirmed in an *in vivo* nude mouse xenograft model of the disease. It was found that ruscogenin induced ferroptosis by regulating the levels of transferrin and ferroportin in pancreatic cancer models, indicating this compound is a potential lead compound against pancreatic cancer.^51^

## 4 Triterpenoid Chemical Probes

The chemical probes in this context are defined as chemical tools that are selective small-molecule modulators of a protein’s function that enable the interrogation of mechanistic and phenotypic questions about its molecular target in biochemical, cell-based, or animal studies.^52^ A good chemical probe engages its target intracellularly and is accompanied by a chemically similar, but inactive, molecule to be used as a negative control in cellular studies. In some cases, chemical probe sets or tool boxes are required to study specific biological processes in disease models.^53^

**Figure 7:**
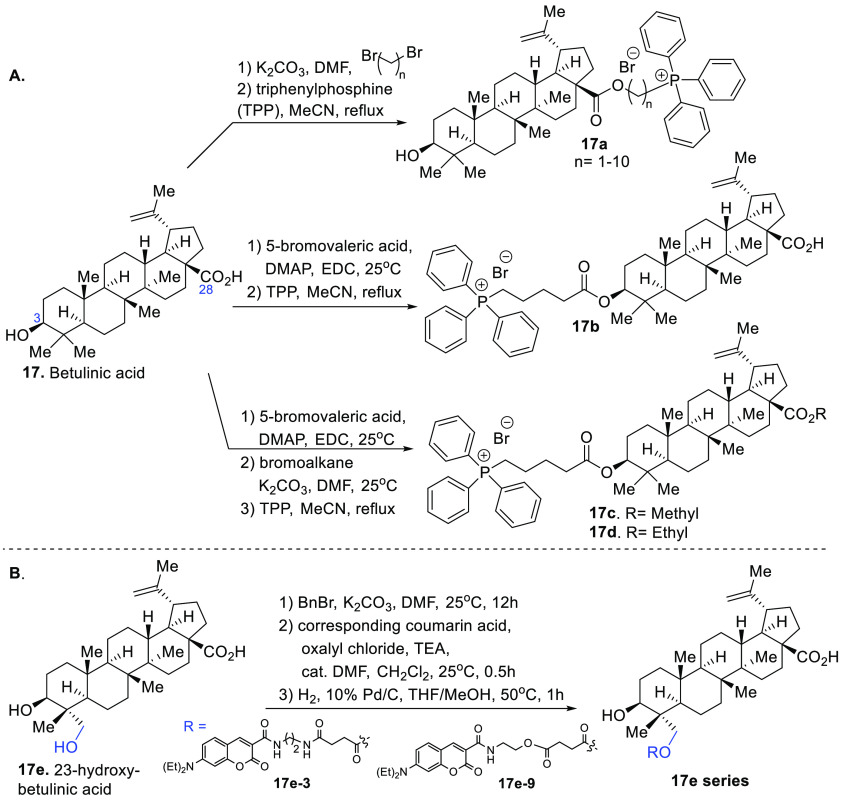
Diverse synthesis of betulinic acid probes.

Although triterpenoids betulin (compound **16**, Figure 1) and betulinic acid (compound **17**, Figure 1) have been widely studied as cancer agents, their mechanism of action is not clear. In addition, these triterpenoids suffer from poor aqueous solubility, low bioavailability, and limited intracellular accumulation capacity, which are unfavorable for further development. Therefore, compound **17**-loaded liposomes consisting of phosphatidylcholine, cholesterol, and mannosylerythritol lipid A were generated to evaluate their effects in HepG2 (human liver cancer cell model).^54^ The study showed the antiproliferative activity of free compound **17** can be significantly improved in the liposome formulation, indicating the physicochemical properties of these triterpenoids pose a barrier in the determination of their true therapeutic value.^54^ Other *in vitro* studies have shown betulinic acid (compound **17**, Figure 1) and its oxidized derivative at C-3, betulonic acid, selectively induce cancer cell apoptosis via the intrinsic mitochondrial pathway accompanied by an increase in mitochondrial membrane permeability, mitochondrial swelling, loss of transmembrane potential ΔΨ_m_, and release of pro-apoptotic molecules. The development of a derivative of compound **17** with a mitochondrial targeting tag at C-28 would promote the opening of the mitochondrial permeability transition pore, increasing apoptosis.^55^ The general triphenylphosphonium cation moiety was strategically synthesized in two steps from compound **17**. Briefly, compound **17** was treated with base, and the α,ω-dibromo alkane in DMF, the resultant product, was purified and treated with excess triphenylphosphine to yield the triphenylphosphonium (TPP) conjugate, compound **17a** (Figure 7). The studies indicate the length of the linker has a strong effect on the compound’s potency. However, the addition of a linker improved the efficacy of compound **17** against breast cancer cell models at least 4-fold. The study strongly indicates that subcellular organelle targeting strategies using bioactive triterpenoids can lead to more potent antiagents, and future murine models are needed to advance these compounds to preclinical studies.^55^

Another study of compound **17** was carried out with cancer cell models and a whole organism model (a zebrafish xenograft model) to understand its anticancer properties. Several probes were synthesized from compound **17** with the TPP-based mitochondria-targeted tag at the C-3 hydroxyl group (compounds **17b**–**d**, Figure 7), and it was discovered that betulin and betulinic acid derivatives were more potent, especially for compound **16**, which exhibited improved cytotoxicity against cancer cells with an appreciable therapeutic window against normal cells.^56^

23-Hydroxy-betulinic acid (compound **17e**, Figure 7B) is a triterpenoid structurally related to compound **17** with the primary difference being the hydroxyl group at C-23. The natural product compound **17e** was isolated from *Pulsatilla chinensis* and has been shown to induce apoptosis via mitochondrial membrane potential depolarization, but its biological targets remain elusive.^57^ To enrich our understanding of mechanism of action of compound **17e**, a set of chemical probes was synthesized, including the use of a diverse linker approach to gain insight into its subcellular accumulation. The coumarin chromophore was chosen as the fluorescent tag due to its versatility of undergoing amidation, alkylations, and esterification at its carboxylic acid group. The synthesis commenced with the protection of the carboxylic acid of compound **17e** with benzyl bromide followed by the addition of the corresponding coumarin acid chloride and removal of the benzyl group via hydrogenation to provide a series of compound **17e** chemical probes (Figure 7B). A cytotoxicity assay (based on MTT dye: 3-(4,5-dimethylthiazol-2-yl)-2,5-diphenyltetrazolium bromide) conducted for these compounds indicated derivative compounds **17e-3** and **17e-9** showed the most promising effects in the submicromolar range; however, the compounds showed a narrow therapeutic index when comparing cancer cells with noncancerous cells. Live cell studies revealed that the resultant compound **17e** fluorescent probes accumulated mainly in the mitochondria, which is agreement with mitochondrial functional studies upon treatment of these compounds. The study highlights the importance of generating a diverse set of probes to identify the most favorable features of a compound of interest; compound **17e** will need further derivatization to identify its exact biological target and develop superior compounds as therapeutic agents against cancer.^57^

**Figure 8:**
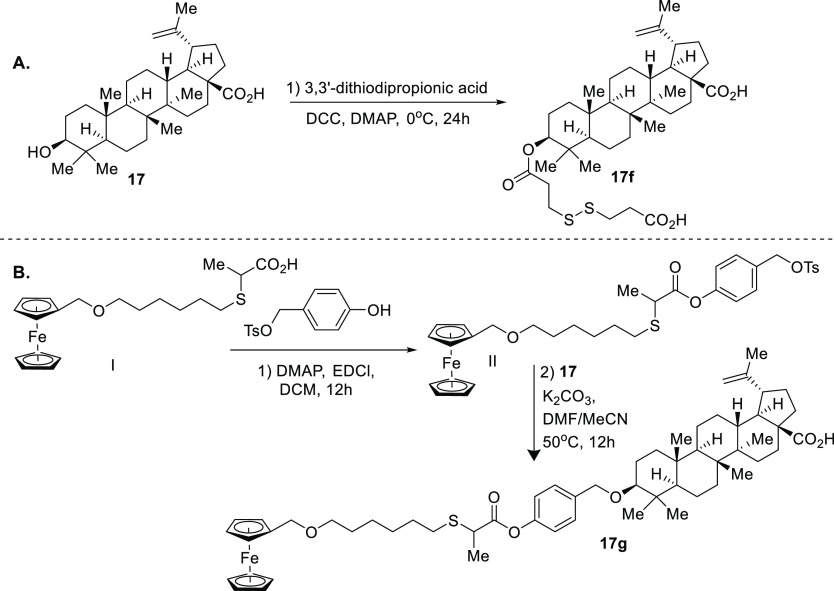
Rapid synthesis to sensing probes of betulinic acid.

While PDT (photodynamic therapy) is a promising therapeutic approach against solid malignancies due to its capacity to generate *in situ* ROS, mediating a cascade of reactions that lead to apoptosis/necrosis, the tumor microenvironment typically overexpresses GSH, a powerful endogenous antioxidant that maintains biological redox balance, scavenging PTD-generated ROS, therefore rendering PTD less effective as an anticancer agent. To overcome this problem, a novel molecular design based on the natural product betulinic acid was developed (compound **17f**, Figure 8).^58^ The synthesis of this chemical probe began with the addition of 3,3′-dithiodipropionic acid to **17** under mild esterification conditions to provide the resultant product in good yield. The glutathione-responsive carrier-free triterpenoid prodrug nano-coassembly of compound **17f** with Ce6 was developed. The nanoparticle resulted in improved singlet oxygen generation and was well tolerated in *in vivo* models. This self-assembled particle displayed a remarkable redox-responsive property, and the *in vitro*/*in vivo* therapy studies demonstrate that they can significantly enhance the synergistic antitumor efficacy with excellent biodegradability and biocompatibility and could be used as a safe photochemotherapeutic agent for potential clinical application and fluorescence imaging. This self-assembly system can serve as a GSH-responsive prodrug nano-coassembly to deliver the chemical cargo at the appropriate subcellular site and improve the antitumor efficacy by reducing excess glutathione. Therefore, it offers a promising therapeutic strategy.^58^

An alternative approach uses a derivative of natural product compound **17** in coassembly with glucose oxidase to generate a novel nanoparticle, which provides a dual attack on the cancer cells.^59^ This study takes advantage of self-encapsulation of organic compounds, **17g** and glucose oxidase, and it also utilizes guest–host interactions between the ferrocene functionality of compound **17g** and the water-soluble pillar[6]arene. The synthesis began with the tosylation of intermediate I to generate intermediate II, which underwent etherification with compound **17** to provide compound **17g**, a thioether tethered ferrocene-modified betulinic acid prodrug. Because this nanoparticle is carrying multiple components, it was expected the release of glucose oxidase would facilitate the generation of gluconic acid and H_2_O_2_, which would facilitate the intracellular Fenton reaction of the thioether tethered ferrocene-modified compound **17g**, significantly increasing the ROS *in situ*. Because cancer cells have higher levels of glutathione, an improved therapeutic index is expected. Overall, this ROS-inducing **17g** was synergistically released with glucose oxidase and promoted the increase of the intracellular ROS level, leading to cancer cell apoptosis. These exciting findings indicate that chemists will continue to use triterpenoids as ROS modulators coupled with other cellular processes to maximize the potential therapeutic outcome.^59^

The natural products ergosterol, ergosterol peroxide (EP, compound **11**, Figure 1), and its related derivative, 9,11-dehydroergosterol peroxide, have been isolated from a variety of fungi and other natural sources.^60−65^ We and others have demonstrated that EP reduces the viability of various cancer cell models, induces caspase-dependent apoptosis through mitochondrial damage via ROS generation,^60−62^ and induces autophagy in various cancer models.^62^ Pretreatment of the cells with the antioxidant NAC minimizes ROS but does not completely abolish EP-mediated apoptosis in those cell models, suggesting other mechanisms are involved in the induction of cell death. While the mode of action of this natural product remains unknown, several hypotheses have been formulated.^63^ Using a yeast model, the progenitor of EP, namely, ergosterol, was shown to undergo peroxidation *in situ* to generate EP as a signaling molecule. Further NMR studies indicated EP inhibited the interactions of master regulator VMS1 (the corresponding human homologue is VCP or valosin containing protein) and CDC48 (human homologue ANKZF1 or ankyrin repeat and zinc finger peptidyl *t*RNA hydrolase 1). VMS1 is translocated to the damaged mitochondria via CDC48 support (a carrier to mitochondria) in response to cellular stress, which generates ROS and mediates the oxidation of ergosterol to EP. Once VSM1 has arrived at the mitochondria, the VSM1/CDC48 complex will support the removal of damaged mitochondria due to accumulated misfolded proteins. While this hypothesis has a strong data set, further mechanistic studies are required for its validation.^63^

**Figure 9:**
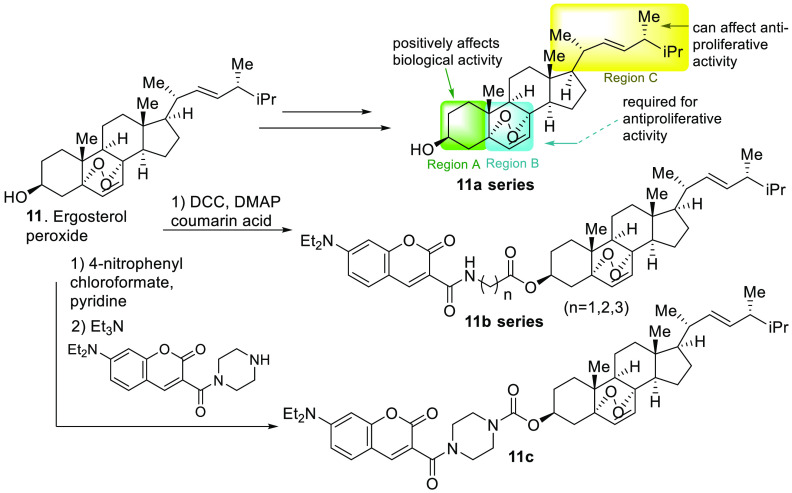
EP chemical probes for structure–activity relationship studies.

An extensive study has been conducted to evaluate the structure–activity relationship (SAR) of EP by various research groups, which has led to the preliminary understanding of the main responsible components of the molecular structure as the three color-coded regions indicate (Figure 9).^64−69^ Region A supports bioactivity as appendages on the hydroxyl group at C-3 can facilitate the solubility properties of this molecule. Region B contains the endoperoxide or warhead required for the main bioactivity observed. Ergosterol has shown modest bioactivity at high dosages (>100 μM)^64^ or in combination with amphotericin B.^65^ The side chain of region C has attracted much attention since it can modulate potency on the basis of the nature of the alkyl group, warranting further investigation. A significant loss of bioactivity was observed with the removal of the side chain, while the medium side hydrocarbon chains promoted bioactivity.^64,66^ While there is accumulated knowledge on the SAR of the EP molecule, less is known about the actual target other than its potential to induce ROS. EP derivatives with a fluorescent tag were reported.^67^ A set of EP derivatives (compounds **11b**,**c**, Figure 9) was generated with the coumarin chromophore. An elaborate series of linkers was constructed with compounds **11b**,**c**, showing the most cytotoxic compounds against cancer cell lines; however, no data was provided on normal cells for comparison. The EP-like compounds inhibit colony formation, migration, and invasion in HepG2 cells. The live cell studies indicate these EP probes accumulate in the mitochondria, enhancing its ROS generating potential.^68^

**Figure 10:**
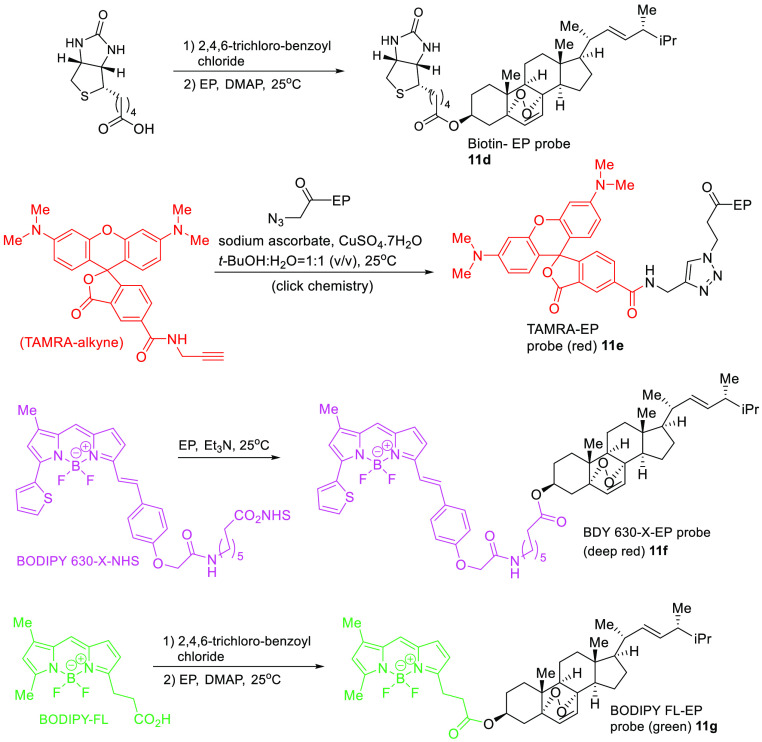
Diverse EP chemical probes to evaluate the mode of action.

A more rigorous report guided by the principle that a set of negative controls was required to conduct washout experiments of the EP probe, and orthogonal fluorescent chromophores had to be synthesized for live cell studies.^69^ Also, the cell lines with fluorescently-tagged organelle were generated to compliment the use of organelle trackers. The hydroxyl group at C-3 of the EP was the direct coupling site for the biotin and chromophores to avoid any further chemical modification of the EP that could interfere with its naive action (compounds **11d**–**g**, Figure 10). Negative controls were synthesized for the three selected fluorescent (FL) tags to conduct parallel studies. First, the red fluorescent tetramethyl rhodamine (TAMRA) was converted to the corresponding terminal alkyne, which could then be coupled with the EP azide via a copper-catalyzed click reaction to produce the triazole compound **11e** in good chemical yields. Probe **11e** presented poor solubility in water and poor cellular permeability; therefore, no live cell information could be collected. However, the Bodipy 630/650 dye, a deep-red fluorescent dye, was coupled with the EP to provide compound **11f** in excellent yields. The boron-based dyes have good physicochemical properties, and the linker geometry and chemical composition can influence the cellular distribution.

Colocalization studies indicated an affinity of compound **11f** toward the mitochondria; however, similar patterns of mitochondrial accumulation were observed for the control dye of compound **11f**. Therefore, the authors generated the shorter linker equivalent by coupling EP with Bodipy FL through esterification directly via the activation of the Bodipy FL carboxylic acid and reacting it with the hydroxyl group of the EP at C-3 to yield compound **11g**. The Bodipy FL dye is bright green with similar excitation and emission to fluorescein and is known for its robust stability. The ER accumulation of compound **11g** was rigorously confirmed by washout experiments (no control probe of compound **11g** remained inside the cell after washout).^69^ Future target validation studies for EP are warranted to facilitate its clinical development.

## 5 Conclusion and Future Perspective

Triterpenoid natural products have served and will continue to serve as critical tools to investigate ROS in living systems. With the overall goal of developing novel therapeutic agents operating via ROS modulation, triterpenoids are a powerful component in the toolbox. This short survey is a tip of the iceberg in terms of the body of knowledge regarding triterpenoid therapeutic potential as the exact mode of action remains elusive. At the cellular level, ROS modulation can lead to cytotoxicity (apoptosis, ferroptosis, autophagy, necrosis, etc.) or cyto-protection (neuroprotection, antioxidant action), defining the cell fate.

Chemical synthesis, semisynthesis, new synthetic methodologies, and biosynthetic gene cluster mining aided by artificial intelligence programs will facilitate access to an array of novel triterpenoids as effective ROS modulators with temporal and spatial control. Further medicinal chemistry endeavors will support rational drug design by revealing potentially new biologic targets of these triterpenoid natural products based on structure–activity relationship and computation studies. Applications of new advanced technologies (PROTACs, molecular glues, CRISPR-cas9) combined with standard techniques, such as EPR (electron paramagnetic resonance spectroscopy) and MALDI-imaging (matrix-assisted laser desorption/ionization time-of-flight imaging mass spectrometry), will close the gap of knowledge in terms of the mode of action of triterpenoids. A better understanding of the role of ROS modulation in cellular signaling will bring us closer to the next generation of personalized medicine.

Some of the key questions to pursue are (a) how do triterpenoids exert the radical initiating step and how do the effects propagate in the cell, (b) are catalysts or cofactors required for ROS modulation, (c) which cellular antioxidants compete with the triterpenoid, and (d) do the triterpenoids support the stability/selectivity of ROS. The answers for these fundamental questions will drive the development of superior triterpenoid tool compounds to better understand ROS in human disease.
